# Reprogramming FGF1 from the natural growth factor to the engineered heparan sulphate biosensor

**DOI:** 10.1186/s12964-025-02269-x

**Published:** 2025-05-28

**Authors:** Krzysztof Ciura, Olimpia Sobota, Aleksandra Chorążewska, Daniel Krowarsch, Natalia Porębska, Łukasz Opaliński

**Affiliations:** 1 Department of Medical Biotechnology, Faculty of Biotechnology, University of Wroclaw, Joliot-Curie 14a, 50-383 Wroclaw, Poland; 2 Department of Protein Engineering, Faculty of Biotechnology, University of Wroclaw, Joliot-Curie 14a, 50-383 Wroclaw, Poland

**Keywords:** FGF1, FGFR, HSPG, Heparin, Heparan sulfate, Protein engineering, Biosensor

## Abstract

Heparan sulphate proteoglycans (HSPGs) are cell surface and extracellular matrix proteoglycan family members, playing a key role in diverse biological activities including signal transduction or endocytosis. To date, many HSPG ligands have been identified, including growth factors interacting via sulfated domains of heparan sulphate (HS) chains. Due to significant overexpression of HSPGs in various cancers, discovering novel biomolecules capable of HSPGs recognition, which may act as HSPGs biosensors or drug carriers targeting HSPGs, is essential. Fibroblast growth factors (FGFs), mainly paracrine FGFs, are natural ligands of membrane-bound HSPGs, forming ternary signaling complex with HSPGs and fibroblast growth factor receptors (FGFRs). Here we employed the natural capability of FGF1 to bind HSPGs to develop HSPGs-specific proteinaceous probe. We identified three point mutations within FGF1 that elevate its affinity for heparin—S116R, S17K and L72R, named B, C and D respectively. Together with substitutions increasing FGF1 stability and abolishing FGF1 binding to FGFRs, we have generated eight HSPGs-specific variants. Among tested mutants, FGF1_HS_BCD exhibits the highest affinity for heparin and HS/HSPGs, showing over 20-fold increase in affinity for glypican-4 and requiring 0.23 M higher salt concentration for elution from heparin column compared to the initial FGF1_HS_ molecule. Finally, we demonstrated FGF1_HS_BCD potential to act as a molecular sensor of HSPGs level in cancer cell lines overproducing HSPGs, implicating that FGF1_HS_BCD can be used as HSPGs biosensor or as a drug delivery carrier in protein-drug conjugates (PDC) targeting HSPGs.

**Supplementary Information:**

The online version contains supplementary material available at 10.1186/s12964-025-02269-x.

## Introduction

Heparan sulphate proteoglycans (HSPGs) constitute family of glycoproteins that are found on the cell surface and in extracellular matrix. HSPGs contain protein core with covalently attached long chains of glycosaminoglycans (GAGs), specifically heparan sulfate (HS), a linear polysaccharide consisting of repeating disaccharide units—uronic acids (glucuronic acid or iduronic acid) and N-acetyl-D-glucosamine, forming N-sulfated and N-acetylated diverse domains [1]. Based on the subcellular localization, three main HSPG groups can be distinguished: membrane-bound/cell surface HSPGs (e.g., syndecans, glypicans), HSPGs of the extracellular matrix (ECM), like perlecan, agrin or collagen XVIII, and the secretory vesicle proteoglycan, serglycin. Serglycin plays an essential role in functions of in the cells of hematopoietic lineage, such as lymphocytes, platelets, and mast cells [2]. As a part of secretory vesicles, serglycin interacting with secretory granule components enables storage and rapid secretion of immunoactive substances at initial stages of immunological processes, for instance, inflammatory reaction [3]. On the other hand, extracellular matrix HSPGs via multiple and functionally independent domains, bind to the other ECM components, defining basement membrane structure [1]. Additionally, ECM HSPGs can bind variety of cytokines and growth factors, presenting those molecules for specific receptors, facilitating signal transduction [4]. Successively, membrane-bound HSPGs are embedded in the plasma membrane in two different ways: syndecans (Sdc1-4) are transmembrane proteins, whereas glypicans (Gpc1-6) are glycosylphosphatidylinositol-anchored proteoglycans. Due to their cell localization, the membrane-bound HSPGs are mainly involved in cell signaling and cell adhesion [4], acting as coreceptors for numerous growth factors, e.g. fibroblast growth factors (FGFs), vascular endothelial growth factors (VEGFs), hepatocyte growth factors (HGFs) [5–8]. Furthermore, membrane-bound HSPGs may act as endocytic receptors, regulate cellular uptake of many secreted and cell surface proteins [1, 9–11]. Importantly, HSPGs overexpression, especially cell surface HSPGs, has been observed in broad spectrum of cancers, such as pancreatic cancer, breast cancer or myeloid leukemia [12–15]. Therefore, there is a strong need for identification of new binding partners of HSPGs that could act as molecular probes for HSPGs or could serve as drug delivery agents in protein drug-conjugate (PDC) strategy.

To date, several monoclonal antibodies (mAbs) specific for particular HSPGs (e.g., glypican-1, syndecan-1, syndecan-4) have been developed. Their capability to selectively recognize HSPGs has been used for detection of HSPGs in biochemistry and molecular diagnostics, and for development of anticancer PDCs in the form of antibody–drug conjugates (ADCs) [13, 16]. Nevertheless, mAbs are specific for selected epitope of protein core of chosen HSPG, which on the one hand ensures their high specificity, but on the other hand doesn’t allow for detection of total HSPGs level. Furthermore, ADCs targeting HSPG are highly vulnerable for deactivation due to cancer cell resistance mechanism, in which expression of particular HSPG target is suppressed and replaced with other member of proteoglycan family.

Natural HSPG ligands, like FGFs recognize HS of all HSPGs, making them universal HSPGs binders. Yet typically natural HSPG ligands also recognize their cognate receptors, like fibroblast growth factor receptors (FGFRs) that together form ternary FGF-HSPG-FGFR signaling complex. Therefore, the high affinity of FGFs for FGFRs limits their specificity for HSPGs. The complex formation between HSPGs and FGFs occurs mainly due to electrostatic interactions between highly negative charges of sulfated HS chains and grouped positively charged amino acid residues in the specific region of FGFs – heparin-binding domain [17]. The pattern of HS sulfation determines specificity and affinity of those interactions [18]. Among all FGFs, paracrine FGFs (e.g. FGF1, FGF2) were identified as the strongest binding partners for HSPGs [19]. So far, two models of molecular assembly of FGF-HSPG-FGFR complex have been proposed—asymmetrical 2:2:1, postulating role of HS as a molecular bridge between two FGF1 molecules and FGFR, resulting in receptor dimerization, and symmetrical 2:2:2 with binding of one HS to one FGF-FGFR complex, inducing dimerization [20, 21]. Nonetheless, the data taken together suggests that both models may occur in solution, supporting the hypothesis of alternative states of FGF-HSPG-FGFR complexes [22]. So far, studies of FGF1 binding to HSPGs have mainly focused on introducing mutations that hamper HS binding, with little information on improving FGF1 affinity for HS or HSPG [23, 24].

In this study we decided to address the question whether it is possible to boost the natural HS-binding properties of FGF1 and uncouple it from FGFR signaling, so that it could act as a molecular sensor of HSPGs.

## Materials and Methods

### Antibodies and reagents

Anti-FGF1 rabbit polyclonal antisera (OMA1) were used as described previously [25]. HRP-conjugated secondary antibodies were obtained from Jackson Immuno-Research Laboratories (Cambridge, UK). Glypican-4 Fc Chimera protein was purchased from R&D Systems, Inc. (Minneapolis, MN, USA). Heparin sodium salt from porcine intestinal mucosa was purchased from Sigma-Aldrich (Saint Louis, MO, USA). Heparin Sepharose 6 Fast Flow affinity resin and the chromatographic column HiTrap Heparin HP was from GE Healthcare (Chicago, IL, USA). All other reagents were obtained from Sigma-Aldrich (Saint Louis, MO, USA).

### Cells

The human osteosarcoma cell line (U2OS) and mouse embryo fibroblast cells (NIH3 T3) were purchased from American Type Culture Collection (ATCC, Manassas, VA, USA), and U2OS cells stably expressing FGFR1 (U2OS-R1) were obtained by transfection of U2OS cells with expression plasmids encoding FGFR1 [26]. U2OS cells were cultivated in DMEM (Biowest, Nuaille, France) supplemented with 10% fetal bovine serum (Thermo Fisher Scientific, Waltham, MA, USA) and antibiotics (100 U/mL penicillin and 100 μg/mL streptomycin). For U2OS-R1 cells, growth media were additionally supplemented with geneticin (0.5 mg/mL) (Thermo Fisher Scientific, Waltham, MA, USA). NIH3 T3 were cultured in DMEM (Thermo Fisher Scientific, Waltham, MA, USA) supplemented with 10% fetal bovine serum (Thermo Fisher Scientific, Waltham, MA, USA) and antibiotics (100 U/mL penicillin and 100 μg/mL streptomycin).

Human pancreatic hTERT-immortalized epithelial cells hTERT-HPNE was purchased from ATCC (Manassas, VA, USA). hTERT-HPNE cell line was cultivated in 75% DMEM without glucose (Sigma-Aldrich, Saint Louis, MO, USA) with additional 2 mM L-glutamine and 1.5 g/L sodium bicarbonate, and 25% Medium M3 Base (Incell Corp., San Antonio, TX, USA), supplemented with 5% fetal bovine serum (Thermo Fisher Scientific, Waltham, MA, USA), 10 ng/ml human recombinant EGF, 5.5 mM D-glucose (1 g/L), 750 ng/ml puromycin and antibiotics (100 U/mL penicillin and 100 μg/mL streptomycin).

Human pancreatic adenocarcinoma cell lines HPAF-II and Capan-2 were purchased from ATCC (Manassas, VA, USA). HPAF-II and Capan-2 cell lines were cultivated in DMEM (Biowest, Nuaille, France) supplemented with 10% fetal bovine serum (Thermo Fisher Scientific, Waltham, MA, USA) and antibiotics (100 U/mL penicillin and 100 μg/mL streptomycin). All cell lines were cultured in a 5% CO2 atmosphere at 37 °C and were seeded onto tissue culture plates one day prior to the start of the experiments.

### Recombinant proteins

The genetic constructs designed for production of FGF1_HS_ and its mutants (FGF1_HS_A, FGF1_HS_B, FGF1_HS_C, FGF1_HS_D, FGF1_HS_AB, FGF1_HS_BC, FGF1_HS_BD, FGF1_HS_CD, FGF1_HS_ABC, FGF1_HS_ABD, FGF1_HS_BCD, FGF1_HS_ABCD) were prepared via gene synthesis (Gene Universal, Newark, DE, USA). Amino acid sequences of produced FGF1_HS_ and its variants are listed in the Supplementary data. The truncated wild-type FGF1 (Met-Ala-FGF1^21–154^ named as FGF1_WT_) cloned in the pET-3c vector [27] was used as a control. To all produced FGF1 proteins, we applied a numbering system according to Gimenez-Gallego et al. (1–140) [28].

Individual colonies of transformed *Escherichia coli* (*E. coli*) BL21(DE3)-RIL strain (Agilent Technologies, Santa Clara, CA, USA) were grown in LB medium with 100 µg/mL ampicillin and 30 µg/mL chloramphenicol for 16 h at 37 °C, 150 rpm. Overnight cultures were then diluted 1:100 in a LB medium with 100 µg/mL ampicillin and 30 µg/mL chloramphenicol and cultivated at 37 °C, 150 rpm to OD_600_ = 0.8. Protein expression was induced by addition of 1 mM IPTG followed by incubation of bacteria at 25 °C for 18 h. After overnight incubation, centrifugation of bacterial cultures was performed (4 °C, 5.000 × g, 10 min), collected bacterial pellet was resuspended in 0.5 M NaCl (in 25 mM HEPES, pH 7.6) and sonicated. After sonication, lysates were centrifuged for 40 min, 14.000 × g in 4 °C. Collected supernatant was incubated with Heparin Sepharose 6 Fast Flow resin (GE Healthcare, Piscataway, NJ, USA) for 2 h or O/N in 4 °C. After incubation, next steps of purification were performed in room temperature. Firstly, resin was washed with 0.5 M NaCl (Wash A) and 0.7 M NaCl (Wash B) (in 25 mM HEPES, pH 7.6) and protein was eluted from the column with a 2 M NaCl (in 25 mM HEPES, pH 7.6). The purity and the identity of the obtained proteins were confirmed by SDS-PAGE, Western blotting and mass spectrometry. Native conformation of the variants was confirmed by circular dichroism and fluorescence spectra measurements.

The extracellular region of FGFR1 fused to the Fc fragment of human IgG1 and Fc fragment of human IgG1 was produced as described previously [29].

### Circular dichroism (CD) measurements

CD spectra were recorded in the wavelength range 200–260 nm in phosphate buffer (25 mM H_3_PO_4_, pH 7.3) at 20 °C on a Jasco J-1500 spectropolarimeter. Spectra were acquired at a protein concentration of 34 µM using a 0.2 mm quartz cuvette.

For thermal denaturation experiments, ellipticity changes at 228 nm were measured at scan rate of 1 °C/min from 20 to 90 °C using 0.5 μM protein samples in phosphate buffer (25 mM H_3_PO_4_, pH 7.3). The data were collected using a 10 mm quartz cuvette on a Jasco J-1500 spectropolarimeter and analyzed using PeakFit software (Jandel Scientific Software) as described previously [30].

### Fluorescence measurements

Fluorescence spectra were measured in phosphate buffer (25 mM H_3_PO_4_, pH 7.3) at 20 °C using an FP-8500 spectrofluorometer (Jasco, Japan) with excitation at 280 nm and emission in the 300–400 nm range. All measurements were carried out at 34 µM protein concentration using a 0.2 mm quartz cuvette.

Thermal denaturation measurements were performed in phosphate buffer (25 mM H_3_PO_4_, pH 7.3) and monitored by measuring the changes in fluorescence emission intensity at 353 nm upon excitation of the single Trp107 at 280 nm. All measurements were carried out at 0.5 µM protein concentration using a 10 mm quartz cuvette. Denaturation data were collected at a scan rate of 1 °C/min from 20 to 90 °C and analyzed using PeakFit software (Jandel Scientific Software) as described previously [30].

### FPLC elution profiles

FGF1 affinity for heparin was evaluated using a HiTrap Heparin-Sepharose column (GE Healthcare) and a linear NaCl gradient (in 25 mM HEPES, pH 7.4) in a range from 0.5 M to 2 M at room temperature, using NGC chromatography system (Bio-Rad, Hercules, CA, USA).

### Western Blotting

Purified proteins or proteins extracted from the cells using a lysis buffer (containing 8% SDS, 2% β-ME), followed by sonication and heating were separated by SDS-PAGE electrophoresis and transferred onto a PVDF membrane using electroblotting. The membrane was probed with a primary antibody that specifically recognizes the protein of interest, followed by washing to remove the unbound primary antibody. To detect the protein–antibody complex, the HRP-conjugated secondary antibody was incubated with the membrane. Finally, the membrane was washed, incubated with HRP-substrate, and the proteins were visualized using ChemiDoc (BioRad, Hercules, CA, USA).

### Far-Western Blotting

To investigate the HSPGs cellular level in different cell lines, Far-Western Blot procedure was performed [31]. Proteins were extracted from the cells using a lysis buffer (containing 8% SDS, 2% β-ME), followed by sonication and heating. Proteins were then separated by SDS-PAGE electrophoresis and transferred onto a PVDF membrane using electroblotting. FGF1_HS_BCD was used as bait protein (5 μg/ml in 2% BSA) for overnight incubation with the membrane at 4 °C followed by incubation with anti-FGF1 primary antibody and HRP-conjugated secondary antibody, and the proteins were visualized using ChemiDoc (BioRad, Hercules, CA, USA). To block the heparin-binding domain of the FGF1_HS_BCD, the bait protein was pre-incubated with heparin prior to incubation with the membrane. Specifically, the bait protein (5 µg/ml) was incubated with heparin (50 µg/ml) in 2% BSA at room temperature for 1 h with gentle mixing. After pre-incubation, FGF1_HS_BCD-heparin complex was used for membrane incubation under the same conditions as described for Far-Western blot procedure.

### BLI measurements

Binding analysis of FGF1_WT_, FGF1_HS_ and its mutant to FGFR1 Fc or glypican-4 Fc Chimera Protein and kinetic measurements of FGF1_HS_ variants to glypican-4 Fc was performed using biolayer interferometry (BLI) with ForteBio Octet K2 (Pall ForteBio, San Jose, CA, USA) in 25 °C with 1000 rpm shaking in Phosphate-Buffered Saline running buffer.

For binding analysis, FGFR Fc or glypican-4 Fc Chimera Protein and the Fc were immobilized on Protein-A sensors (loading step in 150 nM solution for 600 s) in a pairwise manner (studied protein and the Fc as a reference sensor) and sensors were subsequently incubated with studied proteins (400 nM) for 450 s, followed by 450 s dissociation step. Signal for control (the Fc) was subsequently subtracted from the signal of FGFR-Fc and glypican-4 Fc Chimera Protein.

For measurements of binding kinetics, sensor-immobilized glypican-4 Fc Chimera Protein (25 µg/mL, 600 s) was incubated with various concentrations of FGF1 variants (200, 400 and 800 nM) for 450 s, followed by 450 s dissociation step. After incubation with a specific protein concentration, each sensor’s surface was regenerated in 100 mM sodium citrate, pH 3.0, for 30 s. Signal for control was subsequently subtracted from the obtained signal. For measuring the kinetics of FGF1_HS_ variants with heparin, NTA biosensors were used for immobilization of His-tagged proteins (5 µg/mL, 600 s) and sensors were incubated with various concentrations of heparin (0.05, 0.5 and 5 nM) for 450 s, followed by 450 s dissociation step. The heterogeneous ligand (1:1) model was used for data fitting of all measured kinetics using Data Analysis 11 Software (Fortebio).

### Mass spectrometry

Intact protein LC–MS was carried on an M-Class Acquity UPLC system coupled to a Synapt XS HRMS equipped with an ESI ion source interface. Mobile phase A consisted of H2O + 0.1% FA, 0.05% TFA, while mobile phase B consisted of ACN + 0.1% FA, 0.05% TFA. Approximately 2–5 pmol of protein were injected, desalted on-system, and a five minute 20–80% B linear gradient was applied for sample separation on a nanoEase M/Z BEH C4 300 Å, 5 μm, 300 μm x 50 mm column, which was kept at 80 °C. MS data was collected at 1 scan/s through a 300–3000 m/z range in positive polarity and TOF resolution mode. Glufibrinopeptide B solution was acquired in the reference function, and the correction was applied in-acquisition. Raw data was processed using the MassLynx V4.2 software. The protein peak from each run was integrated, the combined spectra was background subtracted, and then deconvoluted using the MaxEnt1 algorithm.

### Fluorescence microscopy

Confocal fluorescence microscopy measurements were carried out using Opera Phenix Plus High-Content Screening system (Perkin Elmer, Waltham, MA, USA). Fixed cells were imaged in confocal mode using 63 × Water, NA 1.15 objective with binning 2 using two peaks autofocus. Images were performed using 2160 × 2160 px Camera ROI, 37 fields per well, with 8–10 Z-stacks per field at 0.5-μm interval to ensure comprehensive imaging of the cell. The Harmony High-Content Imaging and Analysis Software (version 5.1; Perkin Elmer, Waltham, MA, USA) was used for image acquisition and analysis. The number of cells was determined using the DAPI signal, which enables nuclei detection and the Cell Mask Deep Redd signal, which enables cytoplasm detection. Relative spot intensity (RSI) was determined based on signal in Alexa 594 channel (coming from DyLight 550 NHS Ester signal). Endocytosis efficacy was analyzed using normalized RSI (RSI_max_ = 1.0) and statistical analyses were performed using analysis of variance (ANOVA) with Tukey HSD for unequal N (Spjotvoll/Stoline) post-hoc test (*p < 0.05; **p < 0.005 and ***p < 0.001).

## Results

### Design of mutations enhancing FGF1 affinity for HS and HSPGs

**Fig. 1:**
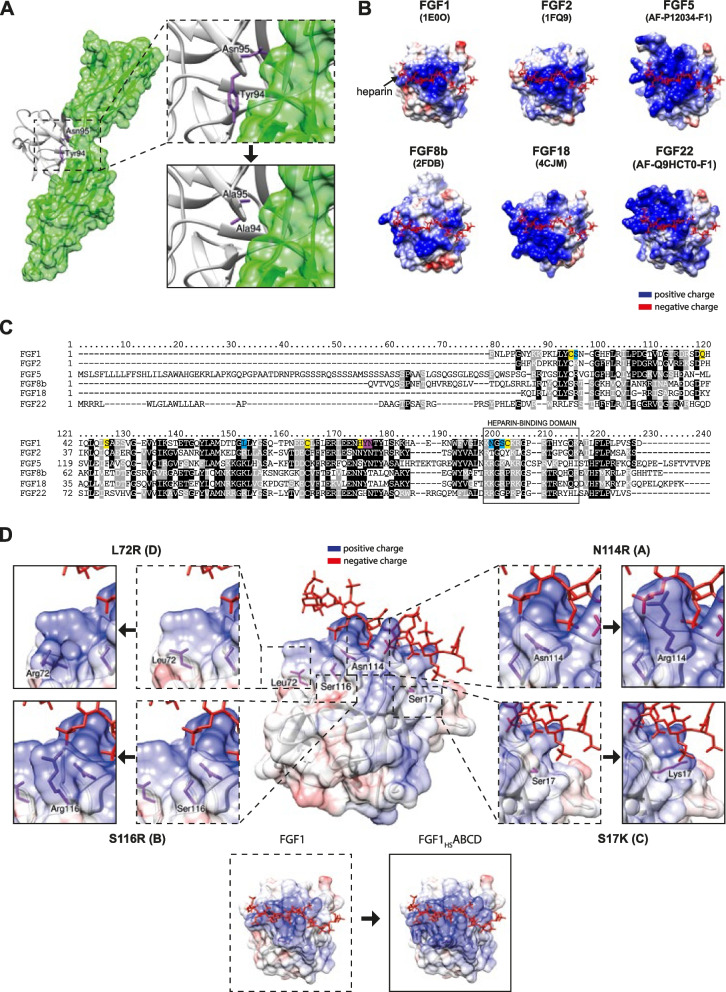
Identification and selection of mutations for FGF1 with increased heparin affinity, increased stability and disrupted FGFR binding. **A.** Molecular surface of crystal structures (FGF1, FGF2, FGF8b, FGF18) and AlphaFold predicted structures (FGF5 and FGF22) of selected FGFs with heparin molecule. If heparin (highlighted in red) was not present in specific structure, heparin from FGF1 crystal structure was implemented to aligned FGF structure. Positive charge was highlighted in blue, negative charge was highlighted in red. **B.** Alignment of selected sequences of FGFs, with highlighted residues chosen for substitutions – heparin affinity mutations (blue), FGFR binding disruption mutations (purple) and stability mutations (yellow). Additionally, on black was highlighted residues repeating at least 3 times in the alignment column, if these residues were not identical but belong to the same amino acid class (polar, nonpolar, positively charged, or negatively charged) these residues were highlighted grey. **C.** Crystal structure of FGF1 interacting with FGFR2 (based on 1E0O) with highlighted residues (purple) for mutations disruptive for FGF1 (white) affinity to FGFR (green) and mutated to alanine residues in Chimera UCSF software. **D.** Molecular surface with positive and negative charge of crystal structure of FGF1 (1E0O.A) with heparin molecule (red). Amino acid residues selected for substitutions increasing heparin affinity (purple) were labeled and mutated using Chimera UCSF software. On the bottom, fully mutated FGF1 (FGF1_HS_BCD) was presented

To fully redirect FGF1 towards HSPGs and enhance its affinity for HS, we have decided to reprogram FGF1 in three different steps. In the first step, we needed to disrupt binding of FGF1 to FGFR. For this purpose, we introduced two well-established point mutations—Y94 A, N95 A, previously shown to interrupt FGFR recognition by FGF1 (magenta label, Fig. 1A and Fig. 1C) [32]. Since molecular sensor for HSPGs should be able to tolerate high temperatures in various applications or techniques, in the second step we decided to improve FGF1 stability by introducing six point mutations: C16S, Q40P, S47I, C83S, H93G, C117S, allowing for increase of melting temperature of wild-type FGF1 for over 20 °C (yellow label in Fig. 1C, Table 1) [30]. FGF1 variant with disrupted binding to FGFR and increased stability has been named FGF1_HS_.

**Table 1:** Introduced mutations increasing HS/HSPG binding, disrupting FGFR affinity and increasing protein stability

**FGF1 mutant**		**Mutation**	
	**↑ HS/HSPG binding**	**↓ FGFR binding**	**↑ Stability**
FGF1_HS_		Y94 A, N95 A	C16S, Q40P, S47I, C83S, H93G, C117S
FGF1_HS_A	N114R	Y94 A, N95 A	C16S, Q40P, S47I, C83S, H93G, C117S
FGF1_HS_B	S116R	Y94 A, N95 A	C16S, Q40P, S47I, C83S, H93G, C117S
FGF1_HS_C	S17 K	Y94 A, N95 A	C16S, Q40P, S47I, C83S, H93G, C117S
FGF1_HS_D	L72R	Y94 A, N95 A	C16S, Q40P, S47I, C83S, H93G, C117S
FGF1_HS_AB	N114R, S116R	Y94 A, N95 A	C16S, Q40P, S47I, C83S, H93G, C117S
FGF1_HS_BC	S116R, S17 K	Y94 A, N95 A	C16S, Q40P, S47I, C83S, H93G, C117S
FGF1_HS_BD	S116R, L72R	Y94 A, N95 A	C16S, Q40P, S47I, C83S, H93G, C117S
FGF1_HS_CD	S17 K, L72R	Y94 A, N95 A	C16S, Q40P, S47I, C83S, H93G, C117S
FGF1_HS_ABC	N114R, S116R, S17 K	Y94 A, N95 A	C16S, Q40P, S47I, C83S, H93G, C117S
FGF1_HS_ABD	N114R, S116R, L72R	Y94 A, N95 A	C16S, Q40P, S47I, C83S, H93G, C117S
FGF1_HS_BCD	S116R, S17 K, L72R	Y94 A, N95 A	C16S, Q40P, S47I, C83S, H93G, C117S
FGF1_HS_ABCD	N114R, S116R, S17 K, L72R	Y94 A, N95 A	C16S, Q40P, S47I, C83S, H93G, C117S

In the third step, we identified possible point mutations increasing FGF1 affinity for heparin. To this end, we compared sequences and structures of FGFs with the strongest affinity for heparin with FGF1 in order to identify non-basic residues in FGF1, which could be substituted for basic amino acid residues (Fig. 1B and Fig. 1C) [33]. We used either crystal structures or AlphaFold-predicted structures of FGFs to evaluate charge composition in heparin-binding domain of these proteins (Fig. 1B). Alignment of all selected FGFs (FGF1, FGF2, FGF5, FGF8b, FGF18 and FGF22) revealed various possible point mutations. However, due to high risk of protein misfolding and steric conflicts, for distinguishing and choosing right positions for substitutions, we had to make at least three assumptions: 1) Lys (K) or Arg (R) residues must be present in the same position at least in three selected FGFs in the alignment; 2) implementation of selected mutation shouldn’t result in steric clashes in 3D structure prediction, 3) substitution should be in the vicinity of heparin to increase probability of interaction. Using such criteria, we identified four best candidates for substitution for lysine or arginine – N114R, S116R, S17 K and L72R, named A, B, C and D respectively (blue label, Fig. 1C and Fig. 1D). Such design led to fully mutated FGF1 variant, FGF1_HS_ABCD presented in Fig. 1D with locally increased positive charges in proximity of heparin in 3D representation of FGF1, achieved due to A, B, C and D mutations.

### ***Isolation and stability analyses of FGF1***_***HS***_*** variants***

**Fig. 2:**
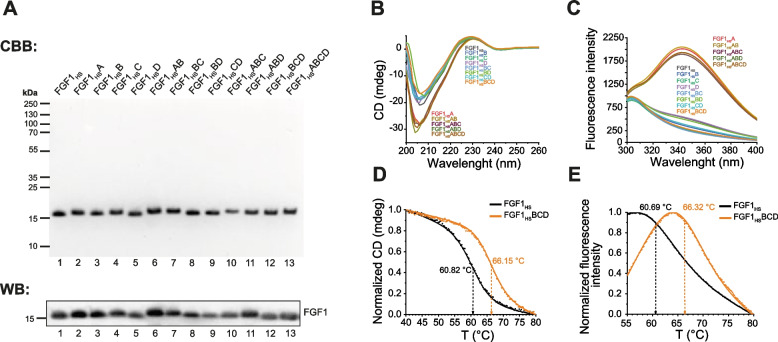
Identification, characteristics and stability of produced FGF1_HS_ variants. **A.** SDS-PAGE and WB of purified all FGF1_HS_ variants. **B.** CD spectra of FGF1_HS_ mutant proteins in 200–260 nm range. **C.** Fluorescence spectra of FGF1_HS_ mutant proteins in 300–400 nm range. **D.** CD thermal denaturation experiments, normalized molar ellipticity at 227 nm was monitored at 20–80 °C. **E.** Fluorescence thermal denaturation experiments, changes in normalized fluorescence intensity were measured at 20–80 °C. For both, CD and fluorescence measurements, scatter plot represents measured points, line represents fitted model

In order to determine the accuracy of designed mutations improving HS binding and to select FGF1_HS_ variant with the highest affinity for HSPG, we prepared a set of twelve FGF1_HS_ mutants with different configurations of specific point mutations identified above (Table 1). All FGF1_HS_ variants were produced in bacterial expression system and purified to homogeneity using affinity chromatography (Fig. 2A, Fig. S1). The identity of purified proteins was confirmed with western-blotting (Fig. 2A) and mass spectrometry (Fig. S2).

Purification of FGF1_HS_ mutants using Heparin Sepharose resin requires native state of isolated proteins, or at least properly folded heparin-binding domain [34]. However, spectral analyses using CD spectrometry demonstrated that all FGF1_HS_ proteins containing N114R substitution (mutation A) showed lower minimum at 207 nm, suggesting, that mutation A leads to conformational changes in FGF1 protein (Fig. 2B). To establish whether FGF1_HS_ variants containing mutation A are partially unfolded, we performed fluorescence scans in 300–400 nm emission range, a common method revealing exposed Trp107 of the hydrophobic FGF1 core of the denatured protein. Fluorescence data demonstrated increased fluorescence emission at 354 nm [35], ultimately confirming that mutation A has caused steric conflicts and probably led to partial protein unfolding (Fig. 2C). Conclusively, we have decided to exclude FGF1_HS_ variants containing mutation A due to possible conformational changes. All other mutants showed CD and fluorescence spectra virtually identical to FGF1_HS_, implicating their proper folding.

Subsequently, thermal denaturation of FGF1_HS_ and its variants was measured using CD and fluorescence spectrometry in order to determine the stability of generated proteins (Table 2, Fig. S3, Fig. S4). These analyses revealed elevated melting temperature of all analyzed mutants in comparison to FGF1_HS_. The highest change was observed for FGF1_HS_ variants containing mutation D, especially for FGF1_HS_BCD variant, with T_m_ increased by about 5.5 °C, depending on used technique (Fig. 2D and Fig. 2E).

**Table 2:** Melting temperature estimation of FGF1_HS_ and selected mutants using CD and fluorescence analysis

FGF1 mutant	Melting temperature (°C)			
	**CD measurements**	**ΔT**	**Fluorescence measurements**	**ΔT**
FGF1_HS_	60.82 ± 1.09	0.00	60.69 ± 0.02	0.00
FGF1_HS_B	61.35 ± 0.07	0.53	62.92 ± 0.04	2.23
FGF1_HS_C	61.10 ± 0.09	0.28	61.70 ± 0.02	1.00
FGF1_HS_D	65.84 ± 0.09	5.02	64.64 ± 0.02	3.95
FGF1_HS_BC	61.95 ± 0.14	1.13	64.53 ± 0.02	3.83
FGF1_HS_BD	65.92 ± 0.03	5.10	64.44 ± 0.06	3.75
FGF1_HS_CD	66.21 ± 0.03	5.39	64.81 ± 0.02	4.12
FGF1_HS_BCD	66.15 ± 0.03	5.33	66.32 ± 0.02	5.63

These data suggest successful generation of FGF1_HS_ variants that, due to implementation of known and novel substitutions, have very high thermal stability.

### Determination of the affinity of developed mutants for HS and HSPG

**Fig. 3:**
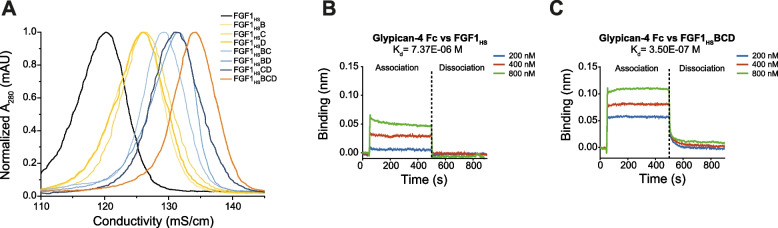
FGF1_HS_ and FGF1_HS_ variants affinity for HS/HSPGs. **A.** Elution profiles of FGF1_HS_ (black), single mutants (yellow), double mutants (blue), and triple mutant – FGF1_HS_BCD (orange) were measured on HiTrap Heparin HP affinity column, with linearly increasing NaCl concentration, directly proportional to conductivity. **B.** BLI estimated binding affinity of FGF1_HS_ with sensor-immobilized glypican-4. **C.** BLI estimated binding affinity of FGF1_HS_BCD with sensor-immobilized glypican-4

To determine whether introduced mutations increased FGF1_HS_ affinity for HS and HSPGs, elution profiles of FGF1_HS_ and FGF1_HS_ mutants were studied using HiTrap Heparin HP column with linearly increasing salt concentration. All developed mutants required higher salt concentration for their elution from the resin in relation to FGF1_HS_, indicting their higher affinity for HS (Fig. 3A). Importantly, this effect has an additive characteristic—the more HS/HSPGs affinity-increasing mutations were introduced to FGF1_HS_, the higher affinity for HiTrap Heparin HP column was observed (Fig. 3A, Table 3).

**Table 3:** Heparin elution profiles and binding affinity of FGF1_HS_ and FGF1_HS_ for glypican-4

FGF1 mutant	Affinity for HS/HSPGs				
	**Elution profile**		**BLI kinetics**		
	**Conductivity (mS/cm)**	**[NaCl] (M)**	**k**_**on**_** (1/µMs)**	**k**_**off**_** (1/s)**	**K**_**d**_** (µM)**
FGF1_HS_	120.19	1.37	0.19 ± 0.04	1.43 ± 0.19	7.37 ± 1.71
FGF1_HS_B	125.86	1.46	0.24 ± 0.01	0.64 ± 0.03	2.64 ± 0.21
FGF1_HS_C	126.34	1.47	0.27 ± 0.01	0.61 ± 0.02	2.27 ± 0.13
FGF1_HS_D	125.89	1.46	0.33 ± 0.02	0.34 ± 0.02	1.01 ± 0.10
FGF1_HS_BC	129.27	1.52	6.83 ± 1.65	0.52 ± 0.12	0.08 ± 0.03
FGF1_HS_BD	131.43	1.55	0.71 ± 0.02	0.30 ± 0.01	0.42 ± 0.02
FGF1_HS_CD	131.06	1.55	0.29 ± 0.01	0.24 ± 0.01	0.81 ± 0.03
FGF1_HS_BCD	134.27	1.60	0.42 ± 0.01	0.15 ± 0.00	0.35 ± 0.01

Using biolayer interferometry (BLI) technique, we measured the kinetics of the interaction between FGF1_HS_ or FGF1_HS_ mutants with the model HSPG—glypican-4 (Table 3, Fig. S5). While FGF1_HS_ interacts with glypican-4 with the K_D_ of 7.37E-06 M, introduced mutations gradually enhanced affinity of FGF1_HS_ for glypican-4, with the triple mutant FGF1_HS_BCD exhibiting a markedly higher affinity (K_D_ = 3.50E-07 M) for glypican-4, representing more than a 20-fold increase in relation to the initial FGF1_HS_ molecule (Fig. 3B and Fig. 3C, Table 3, Fig. S5). Moreover, kinetic measurements revealed that FGF1_HS_BCD displays higher affinity for heparin than FGF1_HS_ (Fig. S6).

These data indicate the successful affinity enhancement of FGF1 for HS and HSPG by all three incorporated mutations. Based on the best binding to heparin/HS/HSPGs and thermal stability characteristics we decided to focus on FGF1_HS_BCD in subsequent studies.

### Assessment of uncoupling of developed FGF1 mutants from FGFR

**Fig. 4:**
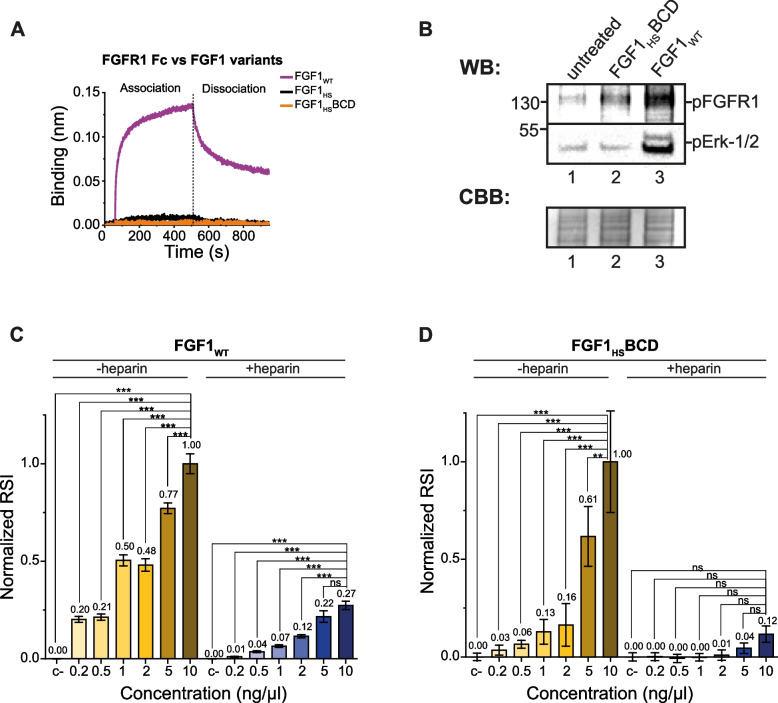
FGF1_WT_ and FGF1_HS_ variants affinity for FGFR, and FGFR/HSPG-dependent endocytosis. **A**. BLI binding analysis of FGF1_WT_, FGF1_HS_ and FGF1_HS_BCD to sensor-immobilized FGFR1. **B.** WB anti-FGFR1 and anti-pErk-1/2 for NIH3 T3 cell lysates supplemented with 20 ng of FGF1_HS_BCD or 20 ng of FGF1_WT_. **C.** Endocytosis efficacy of FGF1_WT_ (increasing concentration in cell media) to U2OS-R1 cells without or with heparin added to cell media, measured by normalized relative spot intensity (RSI) with fluorescence microscopy (RSI_max_ = 1.0). **D.** Endocytosis efficacy of FGF1_HS_BCD (increasing concentration in cell media) to U2OS-R1 cells without or with heparin added to cell media, measured by normalized relative spot intensity (RSI) with fluorescence microscopy (RSI_max_ = 1.0). Average values ± 1.5 SEM are shown. For each group minimum 105 cells were analyzed. Statistical analyses were performed using analysis of variance with Tukey HSD test (*p < 0.05; **p < 0.005 and ***p < 0.001)

To study if introduced mutations successfully uncoupled FGF1 from FGFR binding we used biolayer interferometry (BLI). To this end, we immobilized FGFR1 on BLI biosensor and incubated it with wild type FGF1, FGF1_HS_ and FGF1_HS_BCD. BLI measurements revealed highly efficient binding of the wild type FGF1 to FGFR1 and virtually no interaction between FGF1_HS_ or FGF1_HS_BCD and FGFR1 (Fig. 4A). Next, we tested the impact of FGF1_HS_BCD on FGFR downstream signaling, showing highly decreased level of pFGFR and pERK, indicating reduced FGFR activation upon FGF1_HS_BCD variant supplementation in comparison to FGF1_WT_ (Fig. 4B). We also analyzed specificity of developed molecules for cell surface HSPGs and FGFR1. As a sensitive readout of HSPGs binding we used quantitative measurements of HSPG-dependent endocytosis that takes place after HSPG binding by FGF1 variants. To this end, we fluorescently labelled the wild type FGF1 and FGF1_HS_BCD mutant and incubated tested proteins with model U2OS-R1 cells, which express FGFR1 and HSPGs on their surface. We performed these experiments in the presence or absence of excessive concentrations of heparin that blocks heparin-binding domain of FGF1 and thus prevents interaction of FGF1 with cell surface HSPGs. After brief incubation of tested proteins with model cells, we measured their cellular uptake using quantitative confocal microscopy. As shown in Fig. 4C, although the intracellular signal of FGF1_WT_ decreased to 25.2% in the presence of heparin, the protein was still internalized. In contrast, heparin practically fully abolished the cellular uptake of FGF1_HS_BCD (Fig. 4D).

These data indicate that the wild type FGF1 binds cell surface FGFR1 and HSPGs, and in absence of heparin is internalized mostly via HSPGs. Soluble heparin directs the wild type FGF1 mostly to FGFR1, which is responsible for the uptake of about one fourth of the FGF1. In contrast, FGF1_HS_BCD is deficient in FGFR1 binding, therefore it is internalized exclusively by HSPGs.

### ***FGF1***_***HS***_***BCD act as molecular sensor for HSPGs level in cells***

**Fig. 5:**
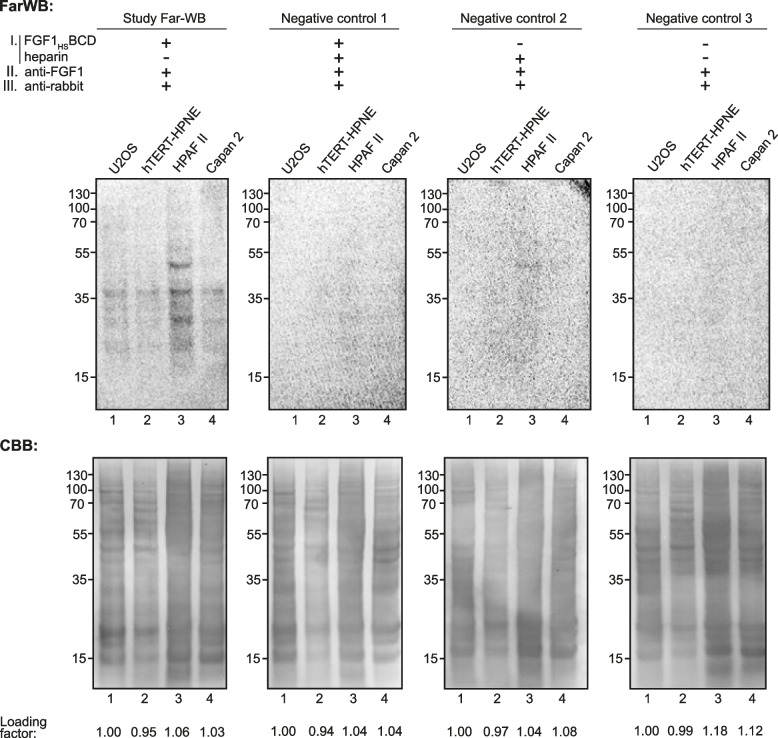
Far-WB analysis of HSPGs level and profile in cells using FGF1_HS_BCD as a bait protein. Study Far-WB and negative controls were performed in presence or absence of FGF1_HS_BCD and/or heparin for the 1 st incubation (top). Far-WB signal analysis (middle) of Study Far-WB and negative controls. Using Image Lab we estimated loading factor (first lane signal equal 1.00) based on loading control of each membrane (bottom) with CBB staining

Due to highly increased affinity for heparin, we assumed that FGF1_HS_BCD will be able to recognize HSPGs in cell lysates and unravel differences between various cell lines, acting as a universal HSPGs molecular sensor. To perform biochemical test evaluating HSPGs level and profiles in different cell lines we decided to conduct Far-WB analysis, using FGF1_HS_BCD as a bait protein. Panel of four epithelial cell lines has been selected including model cell line U2OS, and three pancreatic cell lines – healthy cell line (hTERT-HPNE) and carcinoma cell lines (HPAF-II and Capan-2) reported to overexpress HSPGs [12, 36, 37] (Fig. 5). Far-WB experiments revealed the strongest signal for HPAF-II cell line with the most variable signal profile seen as multiple bands of different molecular weights, especially in the range between 15 to 55 kDa (Fig. 5). Supplementation of FGF1_HS_BCD with soluble heparin resulted in virtually no signal in Far-WB experiments, confirming specific detection of HS in cell lysates by FGF1_HS_BCD (Fig. 5).

## Discussion

We designed and characterized FGF1 variants with strongly enhanced ability to bind HS/HSPGs. Together with simultaneously introduced mutations abolishing binding to FGFR, for the first time we fully reprogrammed FGF1 to selectively recognize HSPGs with the high affinity, generating FGF1_HS_BCD. Our mutants show, that FGF1 affinity to HS can be easily increased by introduction of specific point mutations, based on substitutions for basic amino acid residues in heparin-binding domain or in a close distance to heparin already bound to FGF1. Importantly, our method for discovery mutations increasing FGF1 affinity to HS revealed independently already known S116R (mutation B) and S17 K (mutation C), and undiscovered L72R (mutation D) substitutions. Xia and Blaber showed that S116R mutation has no effect on protein stability [38] and identified its potential for enhancement of FGF1 affinity for heparin [39]. On the other hand, S17 K was found to increase FGF1 affinity for heparin, based on alignment with FGF2 – indicated as S31 K due to different numbering system of FGF1 [24]. To the best of our knowledge, L72R (mutation D) was not identified yet as a mutation enhancing FGF1 affinity for heparin, additionally increasing its thermal stability. Combination of all three described mutations resulted in over 20-fold increase in glypican-4 binding affinity, paving the way for the discovery of further mutations enhancing HS/HSPGs affinity of FGFs.

Conducted BLI experiments and evaluation of endocytosis efficacy of FGF1_HS_BCD to U2OS-R1 cells showed that introduced mutations highly reduced FGF1 affinity for FGFR. Moreover, endocytosis analyses revealed dominant impact of HSPGs on the internalization of FGF1 – our data indicate that HSPG and not FGFR constitute predominant receptors for FGF1 endocytosis. Elimination of FGF1_WT_ interaction with HSPGs resulted in almost 75% decrease in its endocytosis, suggesting that HSPGs might be critical factors in FGF1 signaling, not only by participating in FGF1-FGFR-HSPG signaling complex, but also by fulfilling the role of master regulators of FGF1 endocytosis.

Conclusively, FGF1_HS_BCD was able to recognize numerous HSPGs in cell lysates, acting as a molecular sensor for HSPGs. In contrast to mAb that recognize specific epitope of HSPGs, FGF1_HS_BCD binds selectively to HS, therefore it can recognize the entire cellular pool of HSPGs. Our findings of the highest level of HSPGs with the use of FGF1_HS_BCD biosensor present in pancreatic carcer cell line support previous reports on increased level of HSPGs in pancreatic adenocarcinoma cells and highlight the feasibility of the developed FGF1 variant as HS/HSPG sensor [36, 40–43]. Finally, in comparison to mAb directed against specific HSPGs, FGF1_HS_BCD can be used as a marker of total HSPGs level in cells or tissue, which might be an important parameter in cancer diagnostics [44]. Importantly, due to lack of posttranslational modifications FGF1_HS_BCD can be easily and efficiently produced in bacterial system. This allows rapid and efficient introduction of additional modifications into FGF1_HS_BCD, such as site-specific modifications such with fluorescent dyes.

Modern anticancer strategies employ protein drug conjugates (PDCs) in which highly specific drug carrier (typically mAb) delivers cytotoxic payload to cancer cells expressing specific cell surface receptor [45]. Due to its high specificity for HS/HSPG, ease of manufacturing and chemical coupling of the cytotoxic payload, FGF1_HS_BCD could serve as an attractive drug carrier in PDC approach targeting HSPGs. Further engineering of FGF1_HS_BCD, for example by increasing the valency or stability could be undertaken to additionally boost its affinity for HS and enable highly efficient aggregation dependent endocytosis of FGF1_HS_BCD-based PDC in complex with HSPG [46–48]. Few PDCs in form of ADCs targeting HSPGs have been reported so far [16]. In these PDCs, mAbs are used as a targeting agents, which ensure selectivity, but at the same time are highly vulnerable for loss of action due to changes in HSPGs expression by cancer cells [49]. By recognizing HS of HSPGs FGF1_HS_BCD would be invulnerable to such drug resistance mechanism. [47, 48]

## Supplementary Information

Additional file 1

Additional file 2

## Data Availability

All original, unprocessed data related to this manuscript has been deposited in Zenodo database https://doi.org/10.5281/zenodo.15073211. Until publication access to this link is protected (after publication data will be fully open), yet publisher or reviewers of this journal may receive prior access at any point, upon request.

## Abbreviations

ADC: Antibody-drug conjugate
BLI: Biolayer interferometry
BSA: Bovine serum albumin
CD: Circular dichroism
DMEM: Dulbecco′s Modified Eagle′s Medium
ECM: Extracellular matrix
ESI: Electrospray ionization
FGF: Fibroblast growth factor
FGFR: Fibroblast growth factor receptor
FPLC: Fast Performance Liquid Chromatography
GAG: Glycosaminoglycans
HGF: Hepatocyte growth factor
HRP: Horseradish peroxidase
HS: Heparan sulfate
HSPG: Heparan sulfate proteoglycan
IPTG: Isopropyl β-D-1-thiogalactopyranoside
LC-MS: Liquid chromatography-mass spectrometry
mAb: Monoclonal antibody
PDC: Protein-drug conjugate
PVDF: Polyvinylidene difluoride
RSI: Relative spot intensity
UPLC: Ultra-performance liquid chromatography
VEGF: Vascular endothelial growth factor
WB: Western Blot
