# Supplementary material for: Reprogramming FGF1 from the natural growth factor to the engineered heparan sulphate biosensor
Source: Cell Commun Signal. 2025 May 28;23:248. doi: 10.1186/s12964-025-02269-x (PMC12121223; doi:10.1186/s12964-025-02269-x)
Supplement: Supplementary file 1 — Additional file 1 [file 12964_2025_2269_MOESM1_ESM.docx]

**Supplementary Data**

**
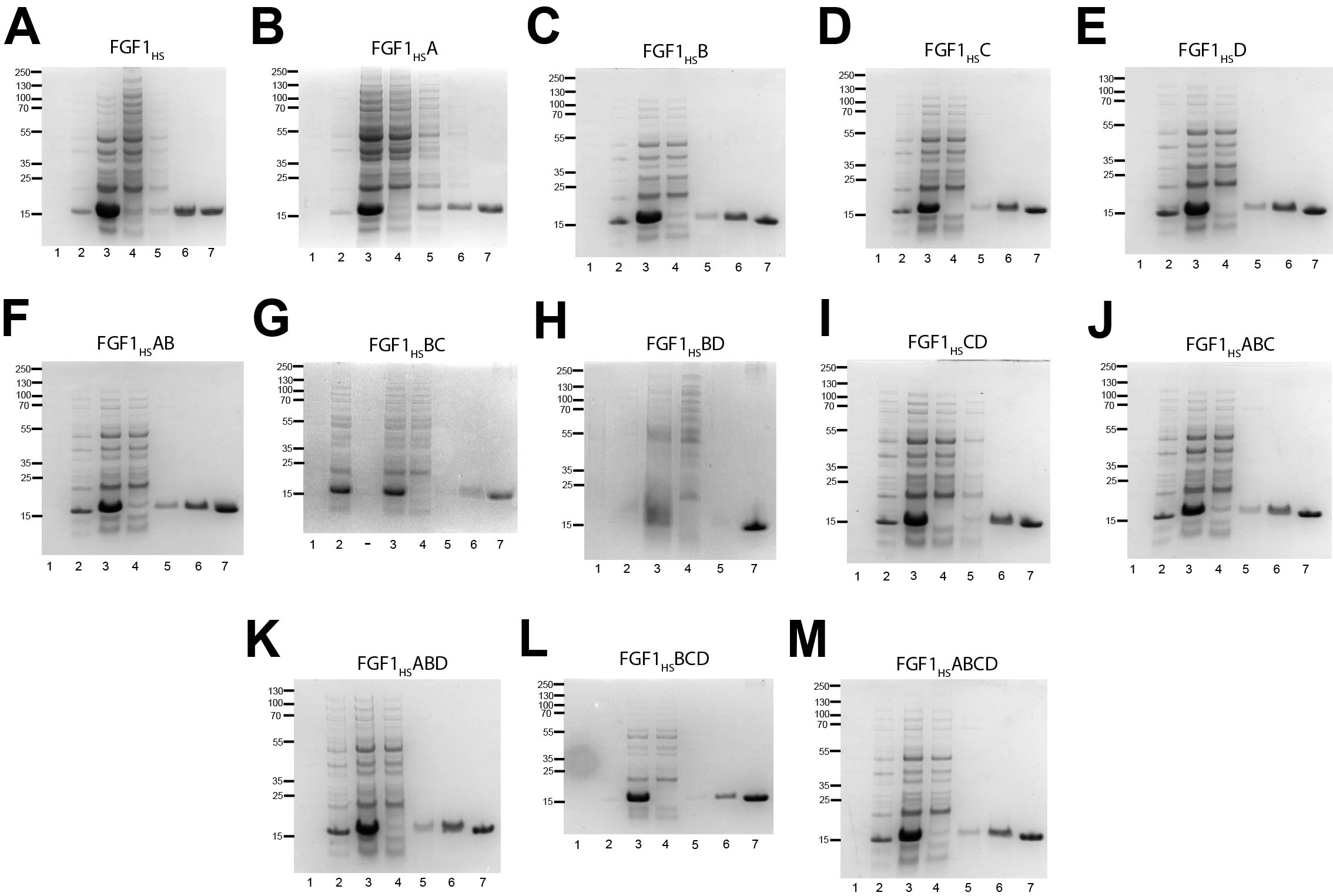
**

**Fig. S1. Overproduction and purification of FGF1_HS_ and FGF1_HS_ variants.** FGF1_HS_ (**A**), FGF1_HS_A (**B**), FGF1_HS_B (**C**), FGF1_HS_C (**D**), FGF1_HS_D (**E**), FGF1_HS_AB (**F**), FGF1_HS_BC (**G**), FGF1_HS_BD (**H**), FGF1_HS_CD (**I**), FGF1_HS_ABC (**J**), FGF1_HS_ABD (**K**), FGF1_HS_BCD (**L**) and FGF1_HS_ABCD (**M**) were purified using Heparin Sepharose 6 Fast Flow resin. 1 – IPTG-, 2 – IPTG+, 3 – supernatant, 4 – flowthrough, 5 – initial wash 0.5 M NaCl, 6 – secondary wash 0.7 M NaCl, 7 – elution 2 M NaCl.


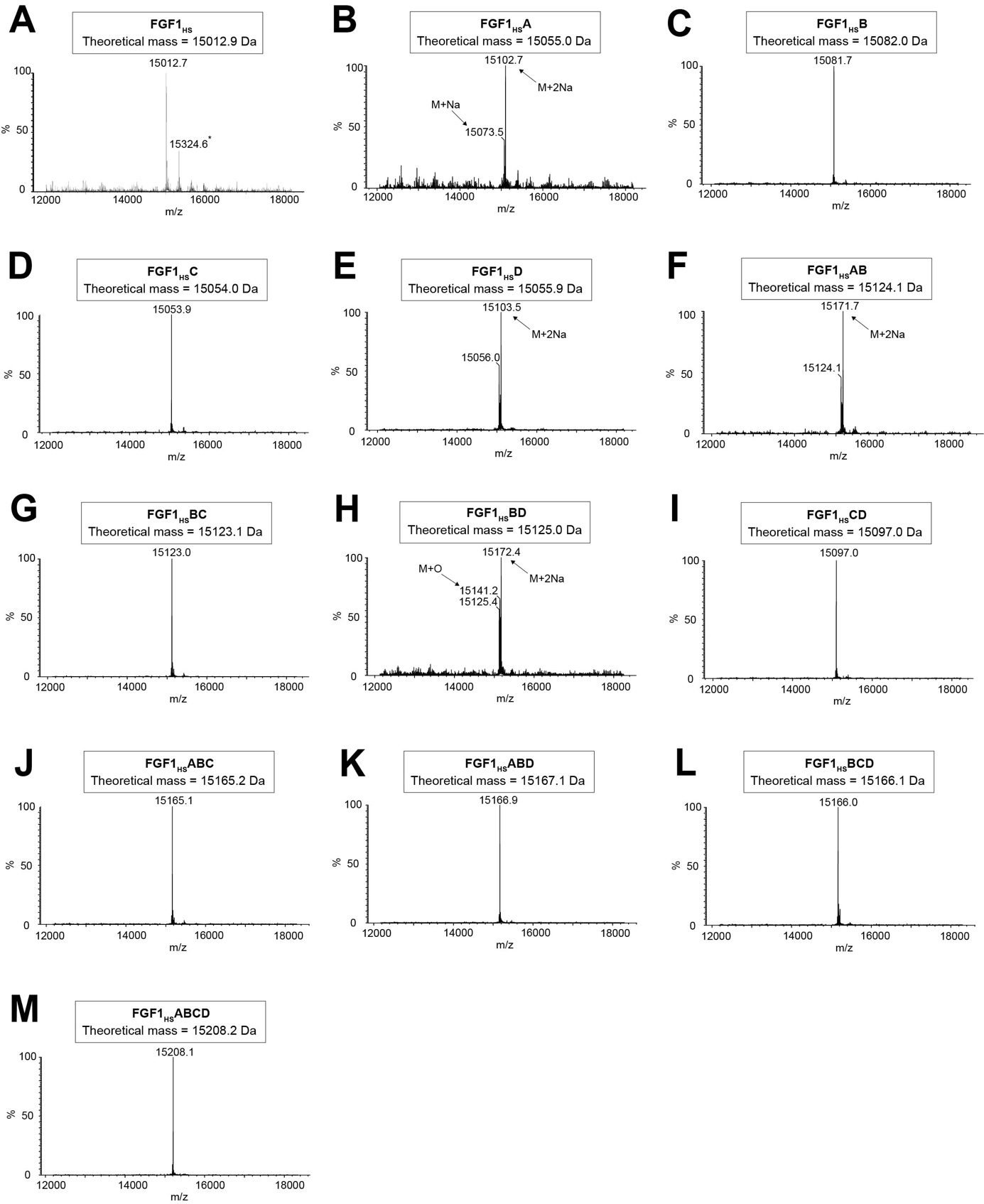


**Fig. S2. Deconvoluted mass spectra** **of FGF1_HS_ and FGF1_HS_ variants using ESI-MS.** FGF1_HS_ (**A**), FGF1_HS_A (**B**), FGF1_HS_B (**C**), FGF1_HS_C (**D**), FGF1_HS_D (**E**), FGF1_HS_AB (**F**), FGF1_HS_BC (**G**), FGF1_HS_BD (**H**), FGF1_HS_CD (**I**), FGF1_HS_ABC (**J**), FGF1_HS_ABD (**K**), FGF1_HS_BCD (**L**) and FGF1_HS_ABCD (**M**). Raw data was processed using the MassLynx V4.2 software. The protein peak from each run was integrated, the combined spectra was background subtracted, and then deconvoluted using the MaxEnt1 algorithm. Peaks correspond to identified neutral masses. Sodium adducts (M+Na and/or M+2Na) and oxidized forms (M+O) are indicated. All measured masses in the MS spectrum are reduced by the mass of methionine due to N-terminal methionine cleavage by methionine aminopeptidase in *E. coli* [1]. * corresponds to protein related mass.

**
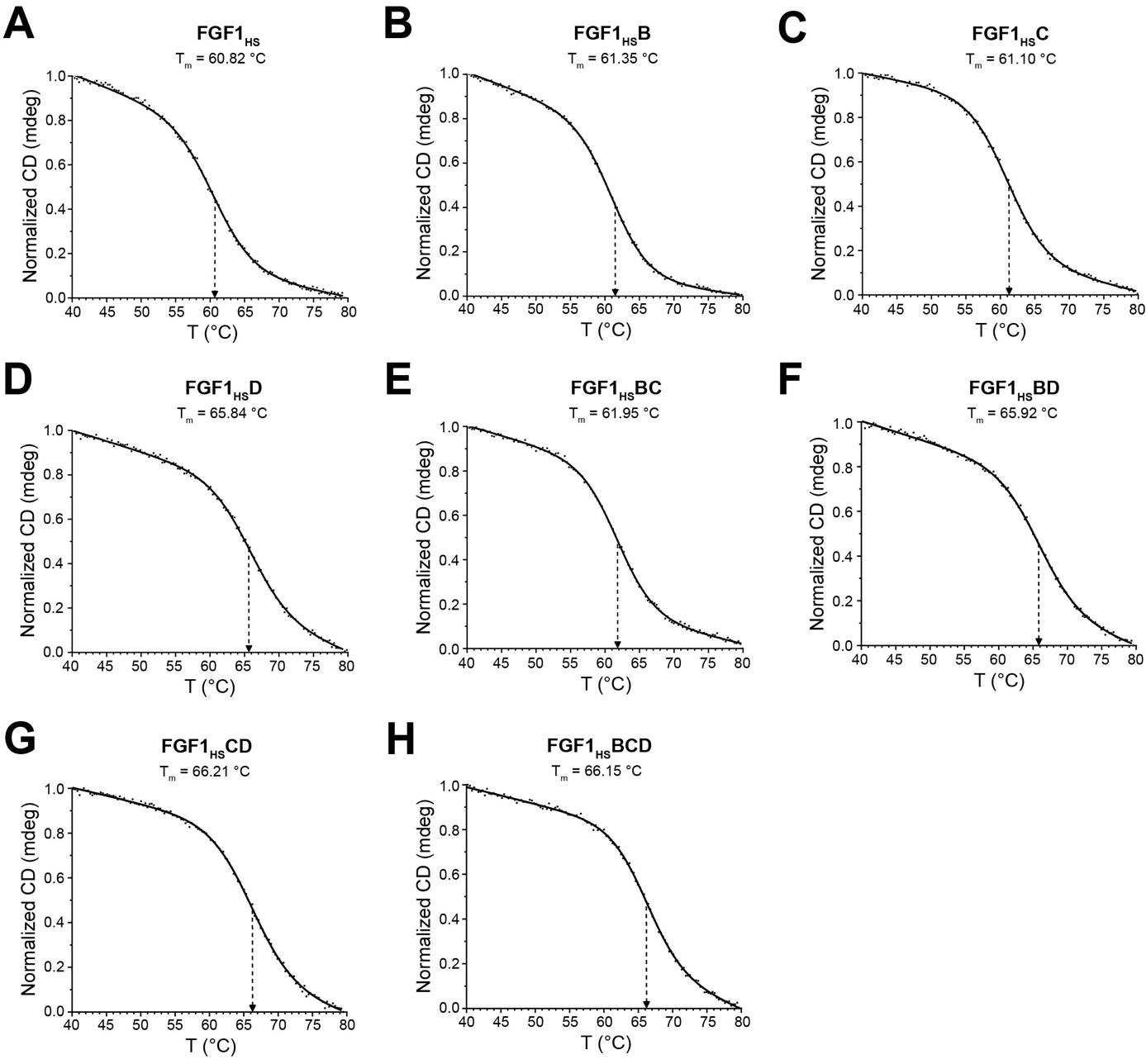
**

**Fig. S3. Estimation of melting temperature for FGF1_HS_ and FGF1_HS_ variants using CD analysis.** FGF1_HS_ (**A**), FGF1_HS_B (**B**), FGF1_HS_C (**C**), FGF1_HS_D (**D**), FGF1_HS_BC (**E**), FGF1_HS_BD (**F**), FGF1_HS_CD (**G**) and FGF1_HS_BCD (**H**). Collected data for each analyzed protein was normalized. The data were collected at a scan rate of 0.25 °C/min and analyzed using PeakFit software (Jandel Scientific Software). Scatter plot represents measured points, line represents fitted model [2].

**
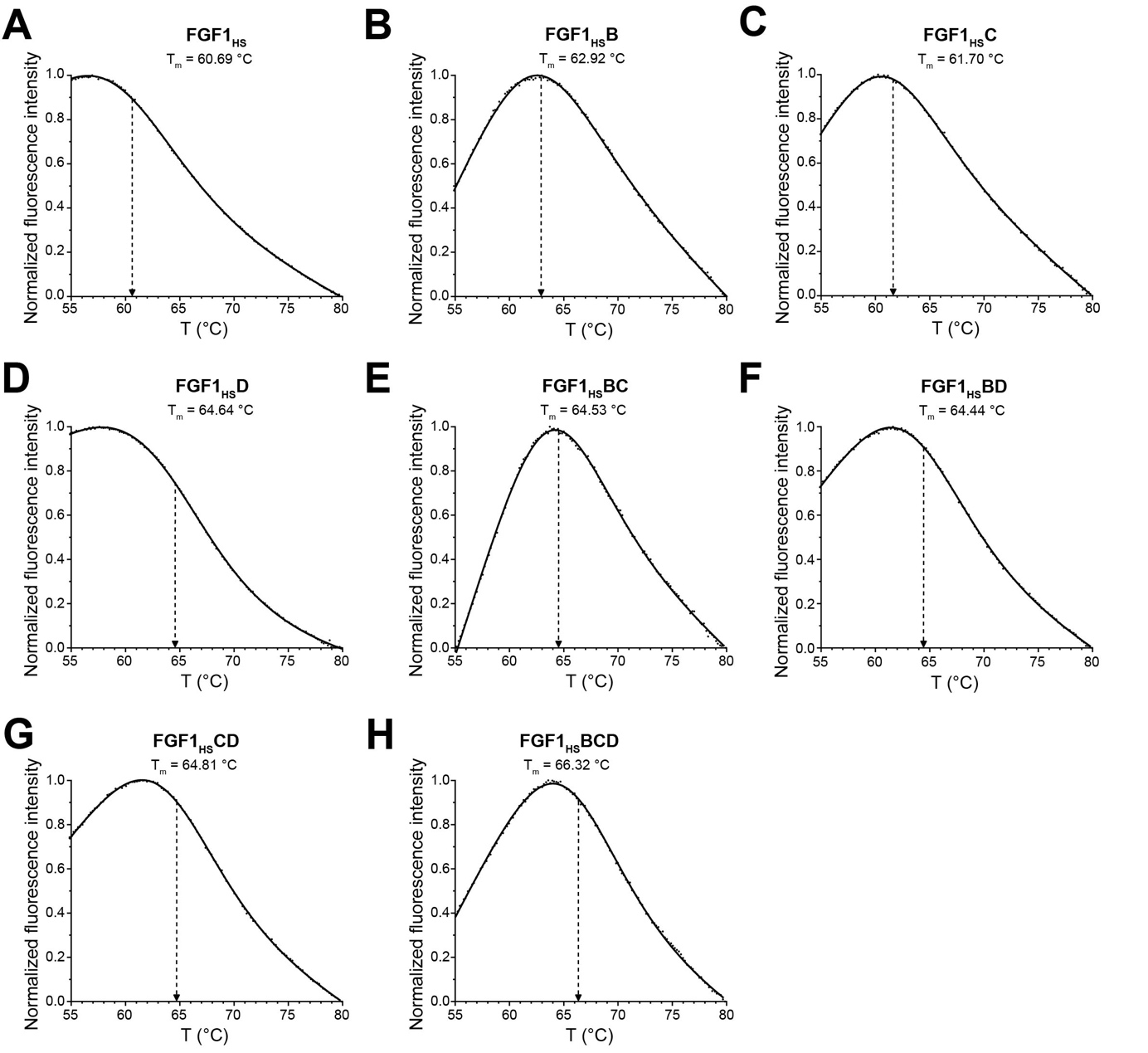
**

**Fig. S4. Estimation of melting temperature for FGF1_HS_ and FGF1_HS_ variants using fluorescence intensity analysis.** FGF1_HS_ (**A**), FGF1_HS_B (**B**), FGF1_HS_C (**C**), FGF1_HS_D (**D**), FGF1_HS_BC (**E**), FGF1_HS_BD (**F**), FGF1_HS_CD (**G**) and FGF1_HS_BCD (**H**). Collected data for each analyzed protein was normalized. Denaturation data were collected at a scan rate of 0.25 °C/min and fitted using PeakFit software (Jandel Scientific Software). Scatter plot represents measured points, line represents fitted model [2].

**
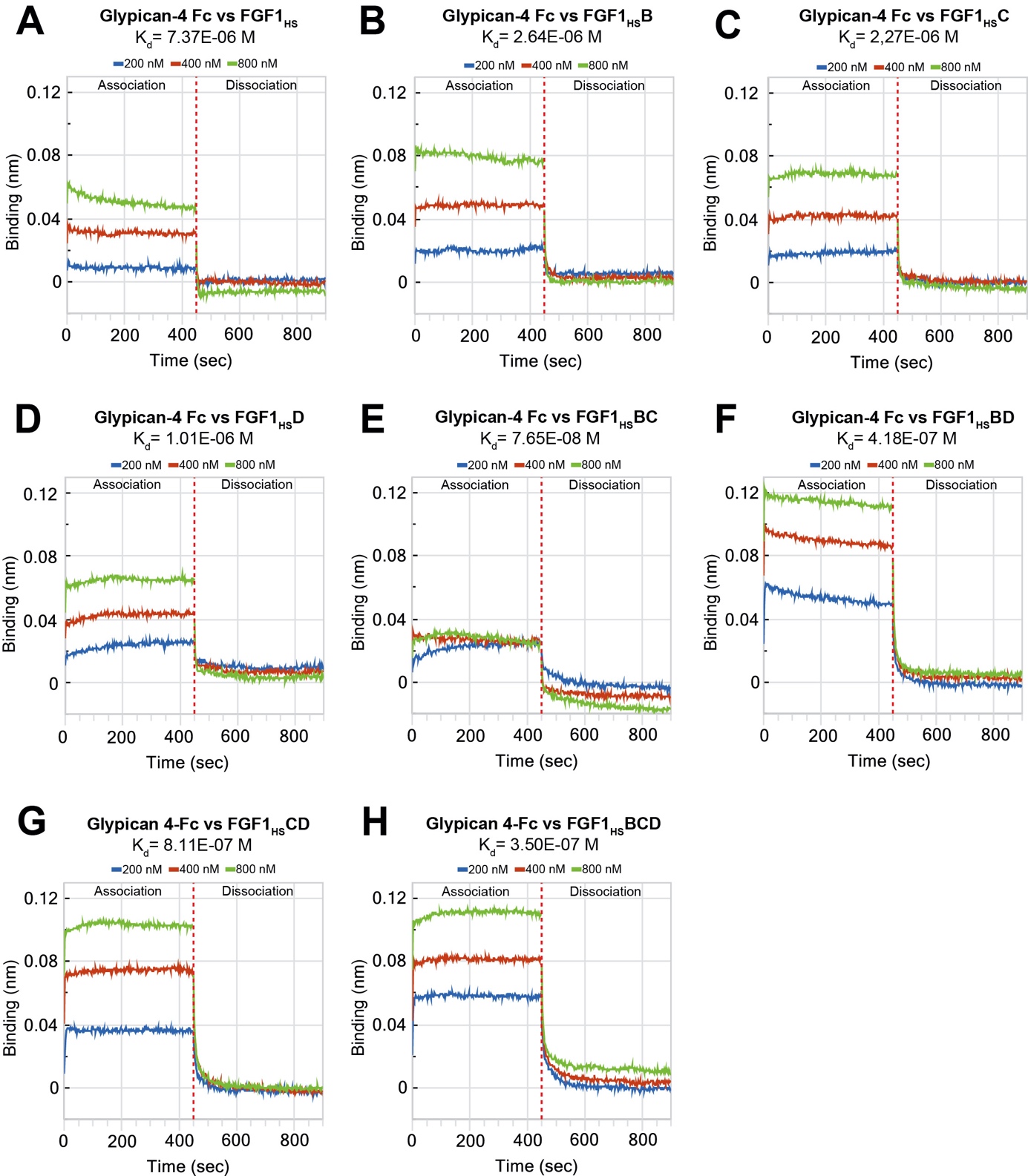
**

**Fig. S5. BLI estimated binding affinity of FGF1_HS_ and its variants with sensor-immobilized glypican 4.** FGF1_HS_ (**A**), FGF1_HS_B (**B**), FGF1_HS_C (**C**), FGF1_HS_D (**D**), FGF1_HS_BC (**E**), FGF1_HS_BD (**F**), FGF1_HS_CD (**G**) and FGF1_HS_BCD (**H**). The heterogeneous ligand (1:1) model was used for data fitting using Data Analysis 11 Software (Fortebio).

**
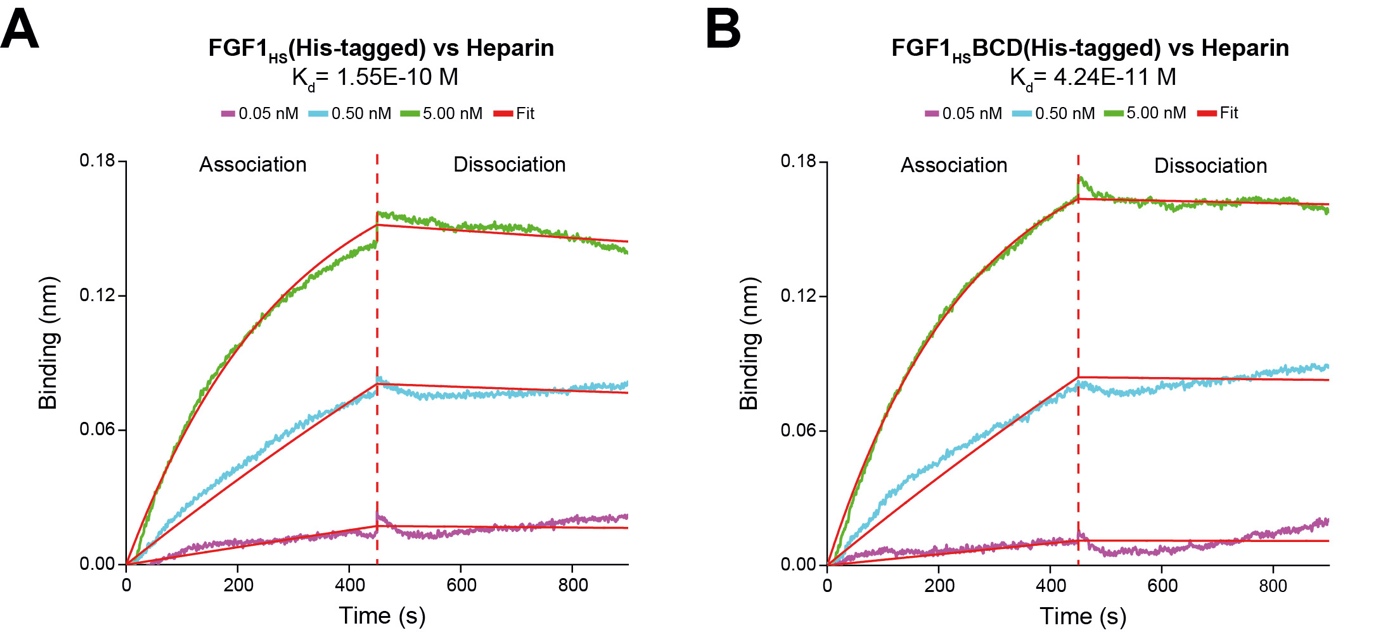
Fig. S6. BLI estimated binding affinity of heparin with sensor-immobilized FGF1_HS_ His-tagged variants.** FGF1_HS_ (**A**) and FGF1_HS_BCD (**B**). The heterogeneous ligand (1:1) model was used for data fitting using Data Analysis 11 Software (Fortebio).

**Amino acid sequences:**

1. **FGF1_HS_** MANYKKPKLLYSSNGGHFLRILPDGTVDGTRDRSDPHIQLQLIAESVGEVYIKSTETGQYLAMDTDGLLYGSQTPNEESLFLERLEENGAATYISKKHAEKNWFVGLKKNGSSKRGPRTHYGQKAILFLPLPVSSD-
2. **FGF1_HS_A**

MANYKKPKLLYSSNGGHFLRILPDGTVDGTRDRSDPHIQLQLIAESVGEVYIKSTETGQYLAMDTDGLLYGSQTPNEESLFLERLEENGAATYISKKHAEKNWFVGLKKRGSSKRGPRTHYGQKAILFLPLPVSSD-

1. **FGF1_HS_B**

MANYKKPKLLYSSNGGHFLRILPDGTVDGTRDRSDPHIQLQLIAESVGEVYIKSTETGQYLAMDTDGLLYGSQTPNEESLFLERLEENGAATYISKKHAEKNWFVGLKKNGRSKRGPRTHYGQKAILFLPLPVSSD-

1. **FGF1_HS_C**

MANYKKPKLLYSKNGGHFLRILPDGTVDGTRDRSDPHIQLQLIAESVGEVYIKSTETGQYLAMDTDGLLYGSQTPNEESLFLERLEENGAATYISKKHAEKNWFVGLKKNGSSKRGPRTHYGQKAILFLPLPVSSD-

1. **FGF1_HS_D**

MANYKKPKLLYSSNGGHFLRILPDGTVDGTRDRSDPHIQLQLIAESVGEVYIKSTETGQYLAMDTDGRLYGSQTPNEESLFLERLEENGAATYISKKHAEKNWFVGLKKNGSSKRGPRTHYGQKAILFLPLPVSSD-

1. **FGF1_HS_AB**

MANYKKPKLLYSSNGGHFLRILPDGTVDGTRDRSDPHIQLQLIAESVGEVYIKSTETGQYLAMDTDGLLYGSQTPNEESLFLERLEENGAATYISKKHAEKNWFVGLKKRGRSKRGPRTHYGQKAILFLPLPVSSD-

1. **FGF1_HS_BC**

MANYKKPKLLYSKNGGHFLRILPDGTVDGTRDRSDPHIQLQLIAESVGEVYIKSTETGQYLAMDTDGLLYGSQTPNEESLFLERLEENGAATYISKKHAEKNWFVGLKKNGRSKRGPRTHYGQKAILFLPLPVSSD-

1. **FGF1_HS_BD**

MANYKKPKLLYSSNGGHFLRILPDGTVDGTRDRSDPHIQLQLIAESVGEVYIKSTETGQYLAMDTDGRLYGSQTPNEESLFLERLEENGAATYISKKHAEKNWFVGLKKNGRSKRGPRTHYGQKAILFLPLPVSSD-

1. **FGF1_HS_CD**

MANYKKPKLLYSKNGGHFLRILPDGTVDGTRDRSDPHIQLQLIAESVGEVYIKSTETGQYLAMDTDGRLYGSQTPNEESLFLERLEENGAATYISKKHAEKNWFVGLKKNGSSKRGPRTHYGQKAILFLPLPVSSD-

1. **FGF1_HS_ABC**

MANYKKPKLLYSKNGGHFLRILPDGTVDGTRDRSDPHIQLQLIAESVGEVYIKSTETGQYLAMDTDGLLYGSQTPNEESLFLERLEENGAATYISKKHAEKNWFVGLKKRGRSKRGPRTHYGQKAILFLPLPVSSD-

1. **FGF1_HS_ABD**

MANYKKPKLLYSSNGGHFLRILPDGTVDGTRDRSDPHIQLQLIAESVGEVYIKSTETGQYLAMDTDGRLYGSQTPNEESLFLERLEENGAATYISKKHAEKNWFVGLKKRGRSKRGPRTHYGQKAILFLPLPVSSD-

1. **FGF1_HS_BCD**

MANYKKPKLLYSKNGGHFLRILPDGTVDGTRDRSDPHIQLQLIAESVGEVYIKSTETGQYLAMDTDGRLYGSQTPNEESLFLERLEENGAATYISKKHAEKNWFVGLKKNGRSKRGPRTHYGQKAILFLPLPVSSD-

1. **FGF1_HS_ABCD**

MANYKKPKLLYSKNGGHFLRILPDGTVDGTRDRSDPHIQLQLIAESVGEVYIKSTETGQYLAMDTDGRLYGSQTPNEESLFLERLEENGAATYISKKHAEKNWFVGLKKRGRSKRGPRTHYGQKAILFLPLPVSSD-

**References**

[1] P. T. Wingfield, “N-Terminal Methionine Processing.,” Curr Protoc Protein Sci, vol. 88, no. 3, pp. 6.14.1-6.14.3, Apr. 2017, doi: 10.1002/cpps.29.

[2] M. Zakrzewska, D. Krowarsch, A. Wiedlocha, S. Olsnes, and J. Otlewski, “Highly Stable Mutants of Human Fibroblast Growth Factor-1 Exhibit Prolonged Biological Action,” J Mol Biol, vol. 352, no. 4, pp. 860–875, Sep. 2005, doi: 10.1016/j.jmb.2005.07.066.
