# Supplementary figures and images for: Reprogramming FGF1 from the natural growth factor to the engineered heparan sulphate biosensor
Source: Cell Commun Signal. 2025 May 28;23:248. doi: 10.1186/s12964-025-02269-x (PMC12121223; doi:10.1186/s12964-025-02269-x)

**
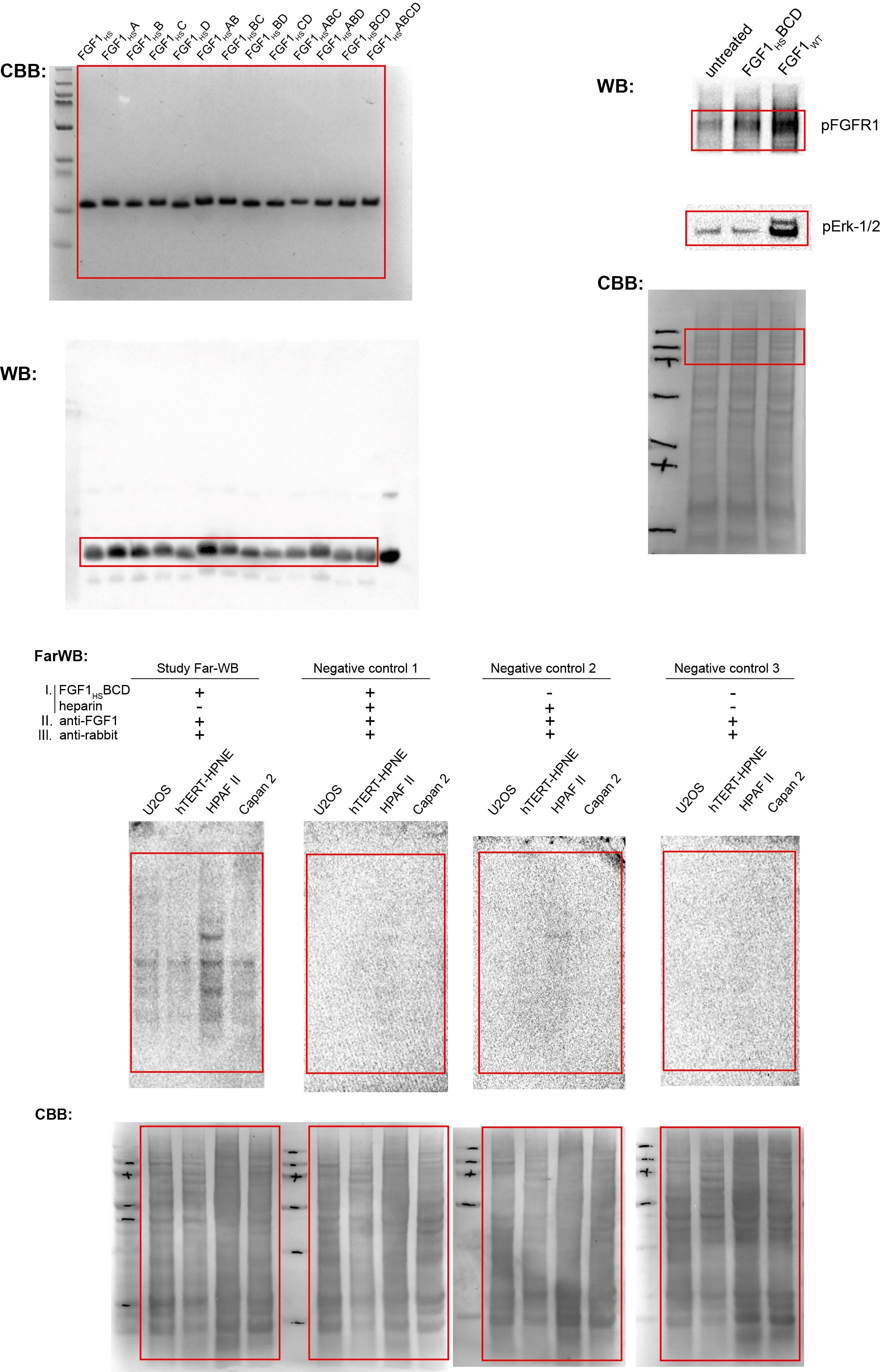
**

**Fig. S. Full length original blots and gels used for preparation of the main figures.**

Supplement: Supplementary file 2 — Additional file 2 [file 12964_2025_2269_MOESM2_ESM.docx]
